# Exchange-free single-pass gateway balloon–assisted neuroform atlas stenting for symptomatic high-grade intracranial atherosclerotic stenosis: clinical and angiographic outcomes

**DOI:** 10.3389/fneur.2026.1854000

**Published:** 2026-06-22

**Authors:** Ismail Karluka, Mustafa Mazıcan, Anıl Tanburoglu, Cagatay Andic

**Affiliations:** 1 Department of Interventional Radiology, Baskent University Dr. Turgut Noyan Application and Research Center, Adana, Türkiye; 2 Department of Neurology, Baskent University Dr. Turgut Noyan Application and Research Center, Adana, Türkiye

**Keywords:** angioplasty, balloon, basilar artery, cerebral angiography, in-stent restenosis, intracranial atherosclerosis, stents, stroke

## Abstract

**Background:**

Symptomatic high-grade intracranial atherosclerotic stenosis (sICAS) is associated with a high risk of early recurrent ischemic events despite optimized medical therapy. Conventional angioplasty-to-stent workflows typically require intracranial exchange maneuvers, which may increase procedural complexity and vessel manipulation in perforator-rich territories of the brain. We evaluated the safety and early angiographic outcomes of a standardized, exchange-free, single-pass Gateway balloon-assisted Neuroform Atlas stenting strategy.

**Methods:**

We retrospectively analyzed consecutive adults with 70–99% sICAS treated at a single center (between January 2018 and January 2025). Following controlled submaximal angioplasty using a Gateway PTA balloon, a Neuroform Atlas stent was deployed directly through the balloon catheter lumen (without microcatheter exchange). Lesion morphology was retrospectively classified according to the Mori system. The primary endpoint was technical success rate. The secondary endpoints were 30-day stroke/intracranial hemorrhage/death and in-stent restenosis (ISR) > 50% on 3-month digital subtraction angiography (DSA).

**Results:**

Fifty-seven patients (median age, 70 years; 68.4% men) were treated after neurological stabilization (median event-to-stent interval, 9 days; range, 7–15 days); 63.2% had posterior circulation targets. By Mori classification, 2 lesions (3.5%) were Type A, 18 (31.6%) Type B, and 37 (64.9%) Type C. The technical success rate was 100%. No flow-limiting dissections, perforations, distal embolizations, acute in-stent thromboses, symptomatic vasospasms, 30-day strokes, or intracranial hemorrhages occurred. The 30-day mortality rate was 1.8% (due to sepsis). Median stenosis improved from 87% pre-procedure to 21.5% post-procedure and was 30% at 3-month DSA (*p* < 0.001). ISR > 50% at 3 months occurred in 7.1% (4/56); cumulative ISR/occlusion during imaging follow-up was 10.5% (6/57), with reintervention in 1.8% of the patients. All early ISR events occurred in the basilar artery lesions. ISR/occlusion rates showed a gradient by Mori type (Type A 0%, Type B 5.6%, Type C 13.5%).

**Conclusion:**

Exchange-free Gateway–Atlas stenting appears technically feasible and was associated with a low observed 30-day neurological complication rate and meaningful luminal gain in a selected cohort of patients with medically refractory sICAS, including a large proportion of morphologically complex (Mori Type C) lesions. The observed basilar artery-predominant restenosis pattern is exploratory and hypothesis-generating, and may justify closer posterior circulation surveillance in future prospective studies.

## Highlights

- What is already known on this topic: Symptomatic intracranial atherosclerotic stenosis (sICAS) carries a high risk of recurrent stroke, despite optimized medical therapy. Endovascular treatment with low-profile self-expanding stents, such as the Neuroform Atlas, has demonstrated improved deliverability compared with that of first-generation platforms. However, conventional balloon-to-stent exchange techniques require intracranial microcatheter exchange after angioplasty, which prolongs instrumentation time and may increase vessel trauma, particularly in the perforator-rich territories.
- What this study adds: In a single-center cohort of 57 carefully selected patients, the exchange-free single-pass Gateway–Atlas technique achieved 100% technical success, no periprocedural neurological complications, no 30-day stroke or intracranial hemorrhage, and one non-neurological 30-day death due to sepsis. Despite a high proportion of morphologically complex lesions (Mori Type C 64.9%, Type B 31.6%), the procedure achieved substantial luminal gain (median stenosis 87 to 21.5%), with ISR > 50% in 7.1% (4/56) at protocolized 3-month DSA and an exploratory cumulative ISR/occlusion rate of 10.5% (6/57) during imaging follow-up. All early ISR events occurred in the basilar artery lesions, suggesting a BA-predominant restenosis signal that requires prospective validation.
- How this study might affect research, practice or policy: These findings support the technical feasibility and low observed 30-day neurological complication rate of exchange-free single-pass stenting as a streamlined endovascular option for carefully selected, medically refractory patients with sICAS. The basilar artery–predominant restenosis pattern suggests that BA lesions may warrant closer postprocedural imaging surveillance. Prospective multicenter studies with standardized longitudinal imaging protocols are required to validate this signal and optimize vessel-specific follow-up strategies.

## Introduction

Symptomatic intracranial atherosclerotic stenosis (sICAS) is a major cause of ischemic stroke. Even with guideline-directed medical therapy, recurrent target territory events are common (1–4). First-generation stent trials, including the Stenting and Aggressive Medical Management for Preventing Recurrent stroke in Intracranial Stenosis (SAMMPRIS) and the Vitesse Intracranial Stent Study for Ischemic Stroke Therapy (VISSIT), showed elevated periprocedural complication rates without a clear benefit over aggressive medical management (1, 2, 5, 6). Endovascular treatment has since been restricted to carefully selected patients who fail medical therapy (1, 7, 8).

Low-profile self-expanding stents, such as the Neuroform Atlas, have improved deliverability and more favorable safety profiles (9–11). However, in-stent restenosis (ISR) and procedural risks have not been eliminated (12, 13). A particular concern is the conventional need for microcatheter exchange after balloon angioplasty, which adds intracranial instrumentation time and may worsen vessel injury (9, 14).

An exchange-free single-pass strategy was designed to circumvent this step. After submaximal angioplasty with the Gateway balloon, the Neuroform Atlas was deployed directly through the same catheter lumen, and no microcatheter exchange was needed (14). In principle, this shortens the procedure, avoids repeated navigation across the diseased segment, and may reduce endothelial trauma in perforator-rich territories (7). However, published data on this technique remain scarce (9, 13, 14). We report our single-center experience with a standardized Gateway–Atlas single-pass protocol, focusing on angiographic outcomes and restenosis patterns in this study.

## Materials and methods

### Study design and patient selection

This single-center, retrospective, observational cohort study was conducted at the Başkent University Dr. Turgut Noyan Application and Research Center and included patients who underwent single-pass intracranial stenting. This study adhered to the Declaration of Helsinki and received ethical approval from the Başkent University Institutional Review Board (March 29, 2022; Project No: KA21/477). Written informed consent for the endovascular procedure was obtained from all the participants. The institutional database was reviewed for patients treated between January 2018 and January 2025.

Endovascular therapy was considered in a multidisciplinary setting involving interventional radiology and neurology teams for patients with recurrent ischemic events despite antiplatelet therapy and vascular risk optimization, in the presence of angiographically confirmed high-grade (70–99%) symptomatic intracranial atherosclerotic stenosis. Eligibility was based on clinical recurrence and angiographic severity; device compatibility was confirmed procedurally, but no otherwise eligible patient was excluded because of vessel anatomy, lesion morphology, perceived technical risk, or operator preference. Although the 90-day window defined the eligibility period for the qualifying ischemic event, procedures were not performed during the acute or hyperacute phase; all patients underwent endovascular treatment only after neurological stabilization, at a minimum of 7 days following the index event.

The inclusion criteria were age ≥18 years, ischemic stroke or transient ischemic attack (TIA) within 90 days, high-grade (70–99%) intracranial stenosis, treatment with a Neuroform Atlas stent delivered through a Gateway PTA balloon catheter, complete procedural data, and postprocedural follow-up. The exclusion criteria were non-atherosclerotic arterial disease, intracranial stenting performed as a rescue strategy during mechanical thrombectomy, alternative endovascular strategies, incomplete documentation, and unavailability of post-procedural follow-up. Patients who met the inclusion criteria and none of the exclusion criteria were included in the study cohort. All consecutive eligible patients during the study period were treated with the exchange-free single-pass technique; no patients were excluded on the basis of vessel anatomy, lesion morphology, perceived technical risk, or operator preference. The only operational exclusion was intracranial stenting performed as rescue therapy during mechanical thrombectomy (*n* = 6), which was kept separate from the present cohort because it represents a distinct clinical and procedural scenario (acute large vessel occlusion with intraprocedural decision-making) that constitutes a separate topic of investigation. No other patients were excluded from the study.

### Endovascular procedure (single-pass technique)

All procedures were conducted under the supervision of C. A., who served as the primary operator in the vast majority of cases. I. K., M. M., and A. T. participated as co-operators or assisting operators as appropriate. The procedures were performed under general anesthesia and sterile conditions at our institution. Vascular access was primarily obtained via the common femoral artery using the Seldinger technique. In a small number of cases, brachial or radial access was used based on the anatomical and technical considerations of the patient. After arterial access, a Neuron MAX 088 Access System (Penumbra, Inc., Alameda, CA, USA) long sheath was positioned in the cervical segment of the parent artery, and a 6F Sofia distal access catheter (MicroVention/Terumo Neuro, Aliso Viejo, CA, USA) was routinely advanced coaxially through it to provide proximal support and stable navigation across tortuous anatomy in all cases. After guide catheter placement, diagnostic cerebral angiography was performed to confirm the lesion location, severity, and vascular anatomy of the target artery. Intravenous unfractionated heparin (5,000 IU bolus) was administered with additional doses to maintain an activated clotting time of 250–300 s during the procedure.

Lesions were crossed using a 0.014-inch hydrophilic-coated Synchro SELECT guidewire (Stryker Neurovascular, Fremont, CA, USA). In all 57 procedures, the lesion was first traversed with the Synchro SELECT microguidewire under roadmap guidance, and the Gateway over-the-wire PTA balloon catheter was then tracked over the microwire to the target lesion; no procedure required conversion to an alternative workflow, and the Gateway balloon successfully reached the target lesion in every case. Controlled submaximal angioplasty was performed with a Gateway over-the-wire PTA balloon catheter (Boston Scientific, Marlborough, MA, USA), which was generally selected to be approximately 50–70% of the reference vessel diameter (measured from the angiographically normal arterial segment immediately proximal to the lesion); the balloon length was chosen to cover the dominant stenotic segment; in diffuse lesions exceeding the available balloon length (maximum 20 mm), segmental balloon angioplasty was performed by sequentially repositioning the balloon along the diseased segment to achieve uniform luminal gain. The balloon was inflated slowly over approximately 60–90 s and inflated slowly up to 6 atm, based on lesion morphology. After deflation, a Neuroform Atlas self-expanding nitinol stent (Stryker Neurovascular, Fremont, CA, USA) was deployed directly through the Gateway balloon catheter lumen, eliminating the need for intracranial microcatheter exchange. Routine post-dilation was not performed in any case; the self-expanding nature of the Atlas stent and the satisfactory immediate luminal gain after submaximal angioplasty obviated additional balloon dilation after stent deployment. The stent size was selected based on the reference vessel diameter and lesion length to cover the diseased segment. Stent sizing followed the manufacturer’s Instructions for Use and the established practice for intracranial atherosclerotic stenosis. The reference vessel diameter was measured on the post-angioplasty angiogram at both the proximal and distal normal arterial segments adjacent to the lesion, and the larger of the two values was used as the reference. The stent diameter was then chosen to match or marginally exceed the larger reference diameter (typically by 0.25–0.5 mm) to achieve adequate wall apposition without aggressive over-expansion. The stent length was selected to cover the diseased segment with at least 4 mm of normal vessel anchoring on each side, accounting for the minimal post-deployment foreshortening of the Atlas device. Stent length reported in Table 1 refers to the longest individual stent implanted; in cases in which the diseased segment exceeded the coverage of a single stent, a second stent was deployed in telescopic fashion with adequate overlap, using the same exchange-free workflow. Final angiography was performed to assess residual stenosis, stent apposition and antegrade flow.

**Table 1:** Baseline characteristics and lesion/procedural parameters.

Variable	Mean ± SD / n/N (%)	Median (min–max)
Demographics		
Age, years	68.84 ± 9.32	70 (34–87)
Male sex	39/57 (68.4)	
Cardiovascular risk factors		
Hypertension	51/57 (89.5)	
Diabetes mellitus	38/57 (66.7)	
Hyperlipidemia	19/57 (33.3)	
Smoking status	Current 11/57 (19.3); Former 24/57 (42.1); Never 22/57 (38.6)	
Clinical presentation		
Qualifying event	Acute ischemic stroke 33/57 (57.9); TIA 24/57 (42.1)	
Baseline NIHSS (at admission)	5.25 ± 3.42	4 (0–15)
Baseline mRS (at admission)	2.93 ± 1.13	3 (0–5)
Qualifying event-to-stent interval, days	—	9 [8–10] (7–15)
Lesion characteristics		
Target artery	BA 18/57 (31.6); V4 18/57 (31.6); ICA-IC 16/57 (28.1); M1 5/57 (8.8)	
Vascular territory	Anterior 21/57 (36.8); Posterior 36/57 (63.2)	
Baseline stenosis (T0), %	85.02 ± 8.65	86 (70–95)
Lesion length, mm	13.96 ± 8.65	13 (4–60)
Mori classification	Type A 2/57 (3.5); Type B 18/57 (31.6); Type C 37/57 (64.9)	
Device parameters		
Gateway PTA balloon diameter, mm	2.84 ± 0.52	2.75 (2–4)
Gateway PTA balloon length, mm	15.88 ± 1.92	15 (15–20)
Neuroform Atlas stent diameter, mm	3.76 ± 0.53	4 (3–4.5)
Neuroform Atlas stent length, mm	25.53 ± 7.84	24 (15–60)
Number of implanted stents	1.07 ± 0.26	1 (1–2)

All values are based on the full cohort (*N* = 57), unless otherwise indicated. Baseline stenosis (T0) is reported here for the full cohort and may differ slightly from values reported in Table 2, which uses the paired imaging cohort (*N* = 56 with available 3-month DSA). Abbreviations: BA, basilar artery; ICA-IC, intracranial internal carotid artery; IQR, interquartile range; M1, middle cerebral artery M1 segment; Mori classification, see reference (18); mRS, modified Rankin Scale; NIHSS, National Institutes of Health Stroke Scale; PTA, percutaneous transluminal angioplasty; TIA, transient ischemic attack; V4, intracranial vertebral artery V4 segment. The IQR was provided only for the qualifying event-to-stent interval.

Dual antiplatelet therapy (ticagrelor 90 mg twice daily plus acetylsalicylic acid 100 mg once daily) was prescribed for 12 months, followed by acetylsalicylic acid 100 mg once daily; the regimen was individualized when clinically indicated. The procedural workflow is illustrated in Supplementary Figure S1.

### Perioperative management

#### Antithrombotic regimen

Because all procedures were performed electively after neurological stabilization (no acute or hyperacute cases were enrolled), dual antiplatelet therapy with ticagrelor 90 mg twice daily and acetylsalicylic acid 100 mg daily was initiated at least 5 days before the procedure in every patient, without the use of loading doses. Platelet function testing (e.g., VerifyNow or P2Y12 reaction units) was not routinely performed. Intraprocedural anticoagulation was achieved with intravenous unfractionated heparin (5,000 IU bolus and additional doses to maintain an activated clotting time of 250–300 s); no postprocedural heparin infusion was administered. The same dual antiplatelet regimen (ticagrelor 90 mg twice daily plus acetylsalicylic acid 100 mg daily) was continued for 12 months after stent implantation, followed by acetylsalicylic acid monotherapy.

#### Blood pressure control

Systolic blood pressure was actively maintained between 120 and 140 mmHg during the periprocedural period and the first 24 h after the procedure to minimize the risk of both ischemic complications and post-procedural hyperperfusion, with intravenous antihypertensive agents administered when clinically indicated.

#### Statin therapy and management of comorbidities

All patients were started on or continued high-intensity statin therapy (atorvastatin 40–80 mg daily). Diabetes mellitus and other established cardiovascular and cerebrovascular risk factors were optimized concurrently according to standard institutional protocols in conjunction with the neurology service.

### Endpoints and definitions

The primary endpoint was the technical success rate. Technical success was defined as successful stent deployment with a post-procedural residual stenosis ≤50% (measured by the Warfarin-Aspirin Symptomatic Intracranial Disease [WASID] (15) method on post-deployment DSA), restoration of antegrade flow, and absence of complications that limited the completion of the procedure.

Periprocedural safety outcomes included cerebrovascular events and mortality occurring during the procedure or within 30 days after the procedure. Operational definitions of safety endpoints were aligned with prior intracranial stenting trials and consensus definitions (4, 16, 17):

- Flow-limiting dissection: angiographic appearance of an intimal flap, intramural contrast staining, or luminal narrowing reducing antegrade flow at the treated segment.
- Vessel perforation/rupture: angiographic extravasation of contrast outside the vessel lumen.
- Distal embolization: new filling defect or branch occlusion distal to the treated lesion on post-procedural angiography.
- Acute in-stent thrombosis: new intraluminal filling defect or occlusion within the stented segment identified before completion of the procedure.
- Symptomatic vasospasm: angiographic vessel narrowing accompanied by clinical deterioration or requiring intra-arterial vasodilator administration.
- Ischemic stroke: new focal neurological deficit persisting >24 h with imaging-confirmed acute infarction in the corresponding territory.
- Intracranial hemorrhage (ICH): any intracranial bleeding identified on CT or MRI, regardless of clinical symptoms; subarachnoid hemorrhage attributable to wire perforation was specifically recorded.
- Hyperperfusion syndrome: post-procedural triad of headache, seizure, or focal deficit with imaging evidence of ipsilateral cerebral edema or hemorrhage and without alternative ischemic explanation.
- Access-site complication: hematoma requiring transfusion or intervention, pseudoaneurysm, or limb ischemia.

All-cause 30-day mortality and non-neurological serious adverse events were recorded.

#### Lesion morphology classification

All target lesions were retrospectively classified according to the Mori classification (18) using preprocedural DSA. Two interventional radiologists independently assigned each lesion to one of three categories: Type A (short, concentric or mildly eccentric lesions with smooth contours and minimal angulation), Type B (tubular or markedly eccentric lesions of intermediate length with moderate angulation), or Type C (long or diffuse lesions, lesions with severe angulation, or lesions located at vessel segments where adjacent perforating branches could not be preserved during dilation). When multiple criteria applied to a single lesion, the most complex feature determined the final category. Discrepancies between readers were resolved by consensus.

Stenosis severity was assessed using digital subtraction angiography (DSA) with the WASID method (15). Two interventional radiologists independently evaluated the angiographic images and reached a consensus on the final measurements; the same approach was used for follow-up imaging to quantify residual and recurrent stenosis.

Angiographic definitions included residual stenosis on post-deployment DSA (WASID), categorized as ≤30% (optimal), 31–50% (acceptable), or >50% (suboptimal) stenosis, respectively. ISR was defined as >50% luminal stenosis within the stent or within 5 mm of the edges (19). Patency was defined as preserved antegrade flow; on CTA/MRA, it was recorded as either patent or occluded. Re-intervention was reserved for symptomatic ISR with recurrent ischemia.

For descriptive completeness, we prespecified non-standard exploratory composite summaries of the short-, intermediate-, and long-term clinical courses. Short-term success was defined as the absence of cerebrovascular events or death within 30 days and the absence of neurological deterioration at the time of discharge. Intermediate clinical composite success was defined as freedom from target-territory recurrent ischemic stroke or TIA through 6 months with a stable functional status. Long-term clinical composite success was defined as freedom from target territory recurrent events beyond 6 months through the last follow-up with a stable mRS.

### Follow-up protocol

Clinical and imaging follow-ups included the National Institutes of Health Stroke Scale (NIHSS) and the modified Rankin Scale (mRS) assessments. Baseline NIHSS and mRS scores were recorded at hospital admission, consistent with our institutional practice; because the median event-to-stent interval was 9 days, baseline values reflected neurological status at presentation rather than immediately before the procedure. Follow-up NIHSS scores were assessed at discharge, and mRS scores were assessed at discharge, 3 months, and 6 months. Digital subtraction angiography (DSA) at 3 months was scheduled for all patients as the reference modality to assess residual stenosis and early in-stent restenosis (ISR) patterns. CT angiography (CTA) at 6 months was performed to evaluate stent patency and antegrade flow, acknowledging the moderate agreement between noninvasive imaging and DSA for intracranial stenosis grading (20). This surveillance schedule reflected our institutional practice for early angiographic assessment rather than a universally standardized imaging protocol for ISR.

Thereafter, clinical follow-up was performed at 6-month intervals. Imaging beyond the scheduled surveillance time points was individualized based on the clinical status, with symptom-driven imaging performed for recurrent neurological symptoms.

In addition, repeat DSA at 12 months post-procedure was planned for all the patients. However, adherence to the planned 12-month DSA was limited in this retrospective cohort; some patients did not reach the 12-month time point owing to death or loss to follow-up, whereas others who reached 12 months underwent CTA or MRA instead of DSA, primarily because of refusal for invasive angiography. When patients developed new or recurrent neurological symptoms, additional vascular imaging (CTA, MRA, or DSA) was performed. Given the heterogeneity in the modality and timing of these examinations, they were not incorporated into the standardized ISR grading. However, for descriptive purposes, an exploratory cumulative imaging endpoint (any ISR > 50% or target lesion occlusion detected on any available DSA, CTA, or MRA during the entire follow-up period) was also reported.

### Statistical analysis

Statistical analyses were performed using R software (version 4.4.1; R Foundation for Statistical Computing, Vienna). Continuous variables are reported as mean ± SD or median [IQR] (min–max), as appropriate, and categorical variables as n (%). Two time-point comparisons for non-normally distributed variables used the Wilcoxon signed-rank test; three or more repeated measures used the Friedman test with Bonferroni-corrected post-hoc comparisons, when appropriate. Categorical variables were compared using Fisher’s exact test. Independent group comparisons for continuous variables were performed using the Mann–Whitney U-test. Given the anticipated low event count, exploratory predictors of ISR/occlusion were assessed using Firth (Jeffreys-prior) penalized logistic regression with a parsimoniously specified *a priori* multivariable model; full model specification and detailed results are provided in Supplementary Tables S1, S2. All tests were two-sided, with *p* < 0.05 considered significant. Missing data were handled using available case analysis.

#### Subgroup analyses

Exploratory subgroup analyses by vascular territory (anterior vs. posterior circulation) and by target vessel (basilar artery vs. non-basilar artery lesions) were performed *post hoc* to assess potential vessel-specific differences in residual stenosis and in-stent restenosis/occlusion. Continuous variables were compared using the Mann–Whitney U test and categorical variables using Fisher’s exact test. Because of the limited number of restenosis events, these analyses were not adjusted for confounders and should be interpreted as hypothesis-generating rather than confirmatory.

## Results

**Figure 1:**
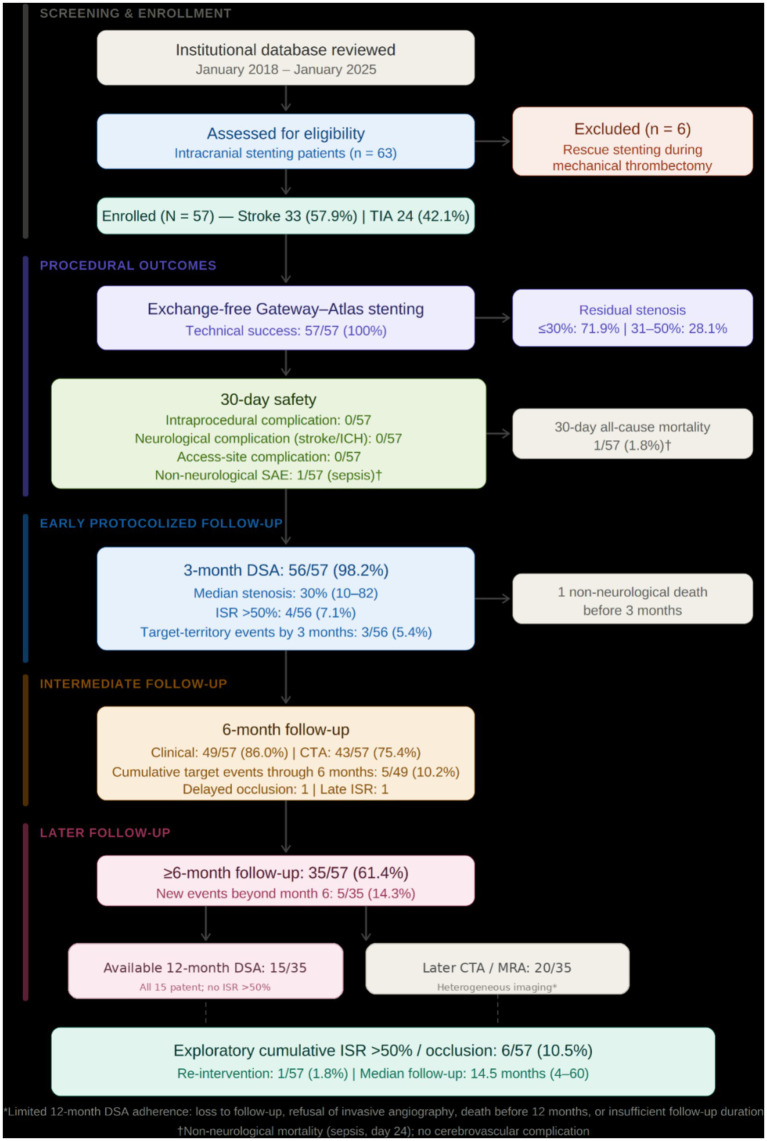
Study flow diagram. Flow diagram illustrating patient screening, enrollment, and follow-up, organized by study phase. Of the 63 patients who underwent intracranial stenting during the study period, six were excluded because stenting was performed as a rescue strategy during mechanical thrombectomy. The remaining 57 patients comprised the study cohort. Procedural outcomes: All patients underwent exchange-free single-pass Gateway–Atlas stenting with 100% technical success, no intraprocedural complications, and no 30-day neurological complications (stroke/ICH); one non-neurological death (sepsis on post-procedural day 24) occurred within 30 days; this is the same patient denoted as the non-neurological serious adverse event in Table 3. Early protocolized follow-up: Three-month digital subtraction angiography (DSA) was completed in 56/57 patients (98.2%), with in-stent restenosis (ISR) > 50% in 4/56 (7.1%) and target-territory ischemic events by 3 months in 3/56 (5.4%). Intermediate available follow-up: Six-month clinical follow-up was available for 49/57 (86.0%) and computed tomography angiography (CTA) for 43/57 (75.4%), with cumulative target-territory ischemic events through 6 months in 5/49 (10.2%). Later available follow-up: 35/57 patients (61.4%) remained under follow-up beyond month 6, all of whom reached the 12-month time point; new target-territory ischemic events beyond month 6 occurred in 5/35 (14.3%) patients. Of these 35 patients, 15 underwent protocolized 12-month DSA (all demonstrating stent patency without ISR > 50%), and 20 were followed up with CTA or magnetic resonance angiography (MRA). Exploratory cumulative ISR > 50% or target lesion occlusion during imaging follow-up was observed in 6/57 (10.5%) patients, with reintervention in 1/57 (1.8%). Because imaging surveillance beyond the protocolized 3-month DSA was heterogeneous and incomplete, this cumulative estimate should be interpreted as being exploratory and potentially conservative. *Of the 57 patients, 22 did not reach 12 months (death, loss to follow-up, or insufficient post-procedural duration); among the 35 who did, 15 (42.9%) underwent protocolized DSA and 20 were followed up with CTA or MRA (primarily refusal of invasive angiography).

Of the 63 patients who underwent intracranial stenting during the study period, six were excluded because stenting was performed as a rescue strategy during the mechanical thrombectomy. No patients were excluded for any other reason, leaving 57 patients in the final cohort (Figure 1). Of the 57 patients, 39 were men (68.4%) with a mean age of 68.84 ± 9.32 years (median, 70; range, 34–87). The presenting conditions were acute ischemic stroke (33/57, 57.9%) and TIA (24/57, 42.1%). The median interval between the qualifying cerebrovascular event and stenting was 9 days (IQR, 8–10; range, 7–15), and no patient underwent stenting during the acute or hyperacute phases. The target vessels included the basilar artery (18/57, 31.6%), V4 segment (18/57, 31.6%), intracranial ICA (16/57, 28.1%), and M1 segment (5/57, 8.8%), with most lesions in the posterior circulation (36/57, 63.2%). By Mori classification, 2 lesions (3.5%) were Type A, 18 (31.6%) Type B, and 37 (64.9%) Type C, indicating that approximately two-thirds of the treated lesions exhibited the most morphologically complex profile. No patient had tandem intracranial or extracranial–intracranial lesions, and all procedures addressed a single target stenosis. In a small number of cases, two stents were telescopically deployed using the same technique to achieve adequate lesion coverage when the stenosis length exceeded single-stent coverage (Table 1).

**Figure 2:**
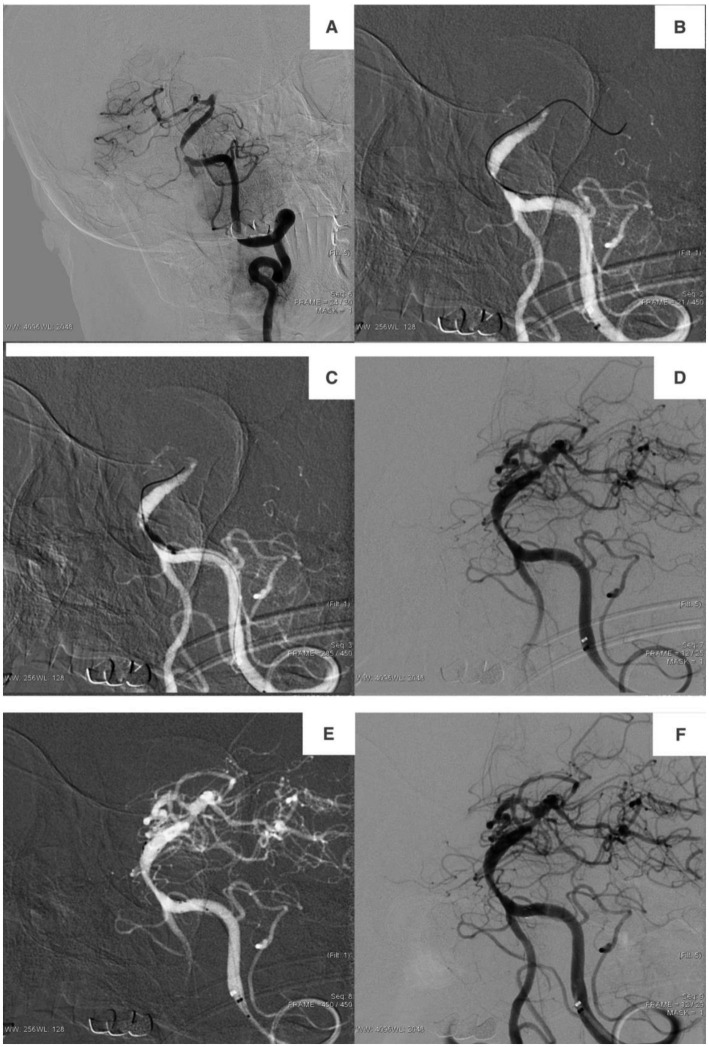
Single-pass angioplasty and stenting of severe basilar artery stenosis. **(A)** Baseline digital subtraction angiography (DSA) of the left vertebral artery reveals a critical (95%) focal stenosis of the basilar artery in a 72-year-old female patient with recurrent posterior circulation ischemic strokes that are unresponsive to medical treatment. The procedure was performed 8 days after the initial event. **(B)** Roadmap-guided navigation of a 0.014-inch microwire was performed across the stenotic segment, facilitating the advancement of the Gateway PTA balloon catheter to the target lesion. **(C)** Submaximal balloon angioplasty was performed under roadmap guidance. **(D)** Post-angioplasty control angiography indicated luminal expansion and enhanced antegrade flow. **(E)** The Neuroform Atlas stent was advanced through the Gateway balloon catheter system and unsheathed across the lesion. **(F)** Final control angiography demonstrates satisfactory stent deployment with acceptable residual stenosis and preserved distal perfusion.

**Figure 3:**
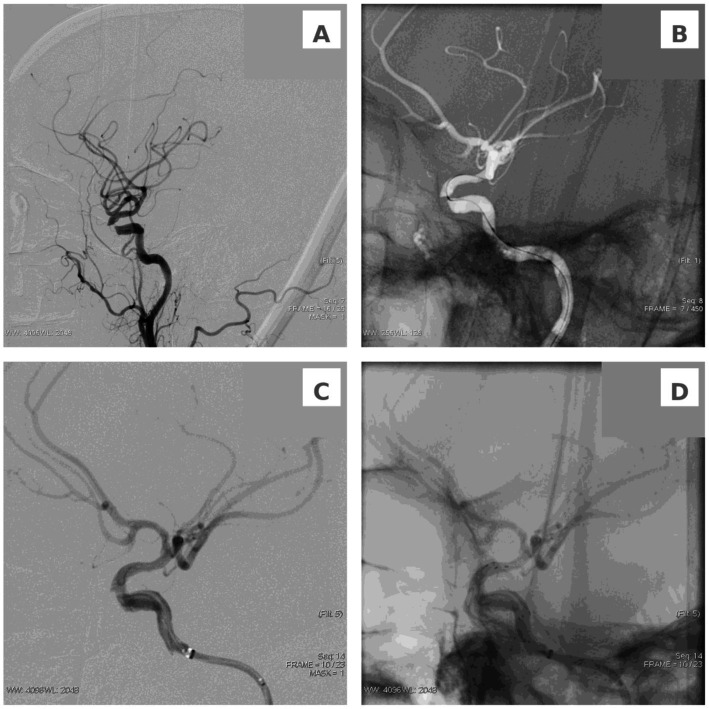
Exchange-free single-pass Gateway–Atlas stenting in a medically refractory intracranial ICA stenosis. **(A)** Lateral digital subtraction angiography (DSA) in a 75-year-old male patient who presented with an initial acute ischemic stroke followed by recurrent transient ischemic attacks despite antiplatelet therapy, revealing significant stenosis in the distal cavernous–paraclinoid segment of the left internal carotid artery. The procedure was performed 9 days after the qualifying ischemic event, following neurological stabilization. **(B)** Roadmap-guided controlled submaximal angioplasty was performed using a Gateway over-the-wire PTA balloon catheter across the stenotic segment. **(C)** The immediate post-angioplasty angiogram indicated enhanced antegrade flow without evidence of flow-limiting dissection, perforation, or distal embolization. **(D)** The final angiogram, following the direct deployment of the Neuroform Atlas self-expanding stent through the Gateway balloon catheter lumen (exchange-free single pass), demonstrates optimal stent expansion and restoration of luminal caliber.

Technical success was achieved in all 57 procedures, with no flow-limiting dissections, vessel perforations, distal embolization, symptomatic vasospasm, or acute in-stent thrombosis. Post-stenting residual stenosis was ≤30% in 41/57 (71.9%) and 31–50% in 16/57 (28.1%); no case had residual stenosis >50%. Representative angiographic cases illustrating basilar artery (Figure 2) and intracranial internal carotid artery (Figure 3) applications of the exchange-free Gateway–Atlas workflow are shown. The mean lesion length was 14.0 ± 8.7 mm. Median stenosis improved from 87% (70–95) at baseline (T0) to 21.5% (0–47) immediately post-procedure (T1), and was 30% (10–82) on 3-month DSA (T2) among the 56 patients with paired imaging; all three time points differed significantly (Friedman test, *p* < 0.001; Table 2, Panel A). Within 30 days, no strokes or intracranial hemorrhages occurred; one non-neurological death due to sepsis was recorded (1/57, 1.8%; Table 3).

**Table 2:** Angiographic outcomes and ISR analysis.

Variable	T0 (pre-procedure)	T1 (post-procedure)	T2 (3-month DSA)	Test statistic	*p*-value†
Panel A. Stenosis (%) across time points (median [min–max])					
Stenosis (%)	87 (70–95)ᵃ	21.5 (0–47)ᵇ	30 (10–82)ᶜ	106.354	<0.001

	T1 residual stenosis category			
	T1 ≤ 30%	T1 31–50%	Total	*p*-value*
Panel B. Association between T1 residual stenosis category and any ISR >50%/occlusion during imaging follow-up (n [%])				
Any ISR > 50%/occlusion present	3 (7.5)	3 (18.8)	6 (10.7)	0.338
Any ISR > 50%/occlusion absent	37 (92.5)	13 (81.3)	50 (89.3)	—

Values are median (min–max) in Panel A and n (%) in Panel B. Panel A repeated-measures analysis included patients with available 3-month DSA (*N* = 56). † Friedman test. Superscripts (ᵃ–ᶜ): time points sharing the same letter do not differ (multiple comparison grouping). * Fisher’s exact test. Panel B analysis included patients with known ISR status (*N* = 56).

**Table 3:** Safety, follow-up, and functional outcomes.

Outcome	n/N (%)
Technical success (stent deployment with residual stenosis ≤50% and preserved antegrade flow)	57/57 (100)
Intraprocedural complication	0/57 (0)
30-day neurological complication (stroke/ICH)	0/57 (0)
30-day access-site complication	0/57 (0)
30-day non-neurological serious adverse event	1/57 (1.8)†
30-day all-cause mortality	1/57 (1.8)†
T1 residual stenosis ≤30% (optimal)	41/57 (71.9)
T1 residual stenosis 31–50% (acceptable)	16/57 (28.1)
T1 residual stenosis >50% (suboptimal)	0/57 (0)
ISR > 50% at 3-month DSA	4/56 (7.1)*
Delayed target-lesion occlusion (month 4)	1/57 (1.8)‡
Late ISR > 50% detected at 6 months	1/57 (1.8)¶
Exploratory cumulative ISR > 50% or occlusion during imaging follow-up (DSA/CTA/MRA)	6/57 (10.5)#
Re-intervention for symptomatic ISR	1/57 (1.8)¶
Target-territory TIA/ischemic stroke by 3 months	3/56 (5.4)*
└ TIA	1/56 (1.8)*
└ Ischemic stroke	2/56 (3.6)*
Cumulative target-territory TIA/ischemic stroke through 6 months	5/49 (10.2)*
└ TIA	3/49 (6.1)*
└ Ischemic stroke	2/49 (4.1)*
6-month available CTA: stent patent/non-occluded	43/43 (100)*
12-month available DSA: stent patent; no ISR > 50%	15/15 (100)*
New target-territory TIA/ischemic stroke beyond month 6	5/35 (14.3)§
└ TIA	1/35 (2.9)§
└ Ischemic stroke	4/35 (11.4)§
Follow-up duration, months, median (min–max)	14.5 (4–60)
Functional outcomes	
NIHSS, median [IQR]	Baseline (admission): 4 [3–8] (*n* = 57); Discharge: 2 [1–4] (*n* = 56)
mRS, median [IQR]	Baseline (admission): 3 [2–4] (*n* = 57); Discharge: 1 [0–2] (*n* = 56); 3 months: 1 [0–2] (*n* = 56); 6 months: 1 [0–2] (*n* = 49)

Values are n/N (%). Denominators reflect available follow-up at each time window. Symbols: † non-neurological 30-day death (sepsis); ‡ delayed target-lesion occlusion at month 4; ¶ late ISR at month 6 (treated with re-angioplasty); # cumulative ISR/occlusion composite (3-month + month-4 + month-6 events); § patients with follow-up beyond 6 months (*n* = 35). The 30-day non-neurological serious adverse event and the 30-day all-cause mortality refer to the same patient (sepsis on post-procedural day 24). The patient with month-4 target-lesion occlusion died before the routine 6-month CTA and was therefore not included in the 43-patient 6-month CTA denominator. Follow-up duration (months) was calculated among patients with available post-procedural follow-up after exclusion of the day-24 sepsis death (*n* = 56); this denominator includes the patient who developed month-4 target-lesion occlusion, with follow-up calculated up to time of death. Operational definitions, complete denominator details, statistical test results (NIHSS Wilcoxon; mRS Friedman with Bonferroni post-hoc), and the 0/57 confidence-interval estimate have been moved to Supplementary Table S5 to streamline this table. Abbreviations: CTA, computed tomography angiography; DSA, digital subtraction angiography; ICH, intracranial hemorrhage; ISR, in-stent restenosis; TIA, transient ischemic attack.

Follow-up availability varied: 3-month DSA in 56/57 patients, 6-month clinical follow-up in 49/57, and 6-month CTA in 43/57 patients. Of the 57 patients, 22 did not reach the 12-month time point owing to death (n = 2), loss to follow-up, or insufficient post-procedural duration. The remaining 35 patients (61.4%) completed follow-up beyond 6 months, and all reached the 12-month time point. Among these 35 eligible patients, 15 (42.9%) underwent protocolized 12-month DSA; the other 20 were followed up with CTA or MRA, primarily because of refusal of invasive angiography. All 15 patients who completed the 12-month DSA demonstrated stent patency without ISR > 50%; however, the low DSA compliance rate among eligible patients (42.9%) and reliance on non-invasive imaging in the remainder may have led to an underestimation of asymptomatic restenosis (Figure 1).

Using available-case denominators at each time window, target territory ischemic events were observed in 3/56 (5.4%) by 3 months, 5/49 (10.2%) cumulatively through 6 months, and 5/35 (14.3%) as new events beyond month 6 among patients with available follow-up beyond month 6 (Table 3).

The ISR > 50%/occlusion rate was 3/40 (7.5%) in the T1 ≤ 30% group versus 3/16 (18.8%) in the T1 31–50% group, without reaching statistical significance (Fisher’s exact test, *p* = 0.338; Table 2, Panel B). Notably, the cumulative ISR/occlusion rate was reported as 6/57 (10.5%) using the full intention-to-treat cohort as the denominator, whereas Table 2 Panel B uses 6/56 (10.7%) among patients with known ISR status, excluding one patient who died before any ISR assessment. Exploratory Firth penalized logistic regression showed nominal univariate associations for hypertension and hyperlipidemia, but no robust independent predictors of any ISR > 50%/occlusion were identified given the very low event count and wide confidence intervals (Supplementary Tables S1, S2). When stratified by Mori classification, cumulative ISR > 50% or target lesion occlusion occurred in 0/2 (0%) Type A lesions, 1/18 (5.6%) Type B lesions, and 5/37 (13.5%) Type C lesions, showing a gradient with increasing morphological complexity (Supplementary Table S4).

Neurological status improved significantly: median NIHSS decreased from 4 [IQR 3–8] at baseline to 2 [IQR 1–4] at discharge (Wilcoxon signed-rank test, *p* < 0.001), and median mRS improved from 3 [IQR 2–4] to 1 [IQR 0–2] at discharge; median mRS remained 1 [IQR 0–2] at both 3 and 6 months, with no significant change between 3 and 6 months (Friedman test on complete cases with data at all four time points [*n* = 49], *p* < 0.001; detailed post-hoc comparisons are provided in Supplementary Table S5).

For descriptive completeness, nonstandard exploratory composite summaries of short-term (56/57, 98.2%), intermediate (44/49, 89.8%), and long-term (30/35, 85.7%) clinical courses were calculated; the single short-term failure was a non-neurological death (sepsis on day 24) in a patient without cerebrovascular complication. In all three windows, the composite rates equalled the event-free rates because no event-free patient met the functional deterioration criterion. These summaries are not validated endpoints and are subject to available case and survivorship bias (see Methods for definitions).

Subgroup analyses by vascular territory and target vessels are presented in Table 4. These comparisons were not adjusted for potential confounders owing to the limited number of ISR events, which precluded meaningful multivariable stratification; therefore, the results should be regarded as exploratory. Baseline and post-stent residual stenosis were comparable between the anterior and posterior circulations (T0: *p* = 0.781; T1: *p* = 0.179) and between the BA and non-BA lesions (T0: *p* = 0.993; T1: *p* = 0.215). At 3-month DSA, early ISR > 50% occurred exclusively in BA lesions (4/17, 23.5% vs. 0/39 non-BA; *p* = 0.006). Two additional post-3-month events contributed to the exploratory cumulative ISR/occlusion endpoint: one delayed target lesion occlusion at month 4 and one late ISR at month 6, both in the posterior circulation. Cumulative ISR > 50%/occlusion was significantly higher in BA than in non-BA targets (5/17, 29.4% vs. 1/39, 2.6%; *p* = 0.008) and showed a non-significant trend toward higher rates in the posterior versus anterior circulation (6/35, 17.1% vs. 0/21, 0%; *p* = 0.074; Table 4).

**Table 4:** Subgroup analysis of restenosis and clinical outcomes.

Variable	Anterior (*n* = 21)	Posterior (*n* = 36)	*p*-value*
Panel A. Vascular territory (anterior vs posterior)			
T0 stenosis (%), median [IQR]	90 [75–95]	85 [80–91.2]	0.781
T1 stenosis (%), median [IQR]	20 [10–30]	24.5 [15–35]	0.179
3-month DSA available, n	21	35	—
T2 stenosis (%), 3-month DSA, median [IQR]	20 [15–40]	30 [21–40]	0.188
ISR > 50% at 3-month DSA, n/N (%)	0/21 (0.0)	4/35 (11.4)	0.286
Target-territory TIA/ischemic stroke by 3 months, n/N (%)	2/21 (9.5)	1/35 (2.9)	0.549
Cumulative target-territory TIA/ischemic stroke through 6 months, n/N (%)	1/16 (6.2)	4/33 (12.1)	1.000
Any ISR > 50%/occlusion during imaging follow-up, n/N (%)	0/21 (0.0)	6/35 (17.1)	0.074

Variable	BA (*n* = 18)	Non-BA (*n* = 39)	*p*-value*
Panel B. Target vessel (basilar artery vs non-basilar)			
T0 stenosis (%), median [IQR]	85 [80–95]	89 [78–92.5]	0.993
T1 stenosis (%), median [IQR]	25 [20–35]	20 [12–32.5]	0.215
3-month DSA available, n	17	39	—
T2 stenosis (%), 3-month DSA, median [IQR]	35 [25–42]	25 [16.5–40]	0.098
ISR > 50% at 3-month DSA, n/N (%)	4/17 (23.5)	0/39 (0.0)	**0.006**
Any ISR > 50%/occlusion during imaging follow-up, n/N (%)	5/17 (29.4)	1/39 (2.6)	**0.008**
Cumulative target-territory TIA/ischemic stroke through 6 months, n/N (%)	3/17 (17.6)	2/32 (6.2)	0.326

*Two-sided *p*-values from the Mann–Whitney U test for continuous variables and Fisher’s exact test for categorical variables. Analyses were based on the available data (missing values excluded). ISR/occlusion and target-territory event rows used available-case denominators reflecting patients with imaging or clinical follow-up at the corresponding time point, not the full intention-to-treat cohort. The 3-month DSA was available for 17 of 18 BA patients; one BA patient died before any follow-up imaging could be performed and was therefore excluded from the ISR/occlusion analysis (BA denominator 17 in the outcome rows). BA, basilar artery; DSA, digital subtraction angiography; IQR, interquartile range; ISR, in-stent restenosis; TIA, transient ischemic attack. Bold values indicate statistically significant differences (*p* < 0.05).

Within BA lesions with available 3-month DSA, post-stent residual stenosis and lesion/device dimensions were similar in lesions with and without early ISR (Supplementary Table S3).

## Discussion

We treated 57 consecutive patients with high-grade (70–99%) sICAS using a standardized exchange-free single-pass Gateway–Atlas protocol between January 2018 and January 2025. By Mori classification, 64.9% of treated lesions were Type C and 31.6% were Type B, indicating that the cohort consisted predominantly of morphologically complex lesions. No procedure was performed in the acute or hyperacute window; all patients underwent stenting after neurological stabilization at a median of 9 days (range, 7–15) following the index event. Technical success was achieved in all 57 procedures performed. No patient experienced stroke or intracranial hemorrhage within 30 days, and the only periprocedural death was unrelated to the nervous system (sepsis; 1.8%). Median stenosis decreased from 87% pre-procedure to 21.5% immediately afterward and was 30% on 3-month DSA (*p* < 0.001), with all post-stent residual values ≤50% (71.9% ≤ 30%; 28.1% 31–50). ISR > 50% was observed in four of 56 patients (7.1%) on 3-month DSA, rising to six of 57 (10.5%) over the entire imaging follow-up, with reintervention required in only one patient (1.8%). Given the heterogeneity of imaging surveillance beyond the protocolized 3-month DSA, and particularly the fact that only 15 of 57 patients completed 12-month DSA, these figures should be interpreted with appropriate caution.

First-generation randomized trials reported substantially less favorable periprocedural outcomes: SAMMPRIS reported a 14.7% rate of stroke or death within 30 days in the stenting arm (21), while VISSIT reported a 24.1% rate of the primary 30-day safety endpoint (stroke or transient ischemic attack within the territory of the qualifying artery, or hard cerebrovascular endpoint) in the stenting arm, of which approximately 14.6% were attributable to periprocedural strokes, with neither trial demonstrating superiority over aggressive medical therapy (1, 3, 16). More recently, the China Angioplasty and Stenting for Symptomatic Intracranial Severe Stenosis (CASSISS) trial, a multicenter randomized comparison of stenting plus medical therapy versus medical therapy alone in 358 patients, with enrollment restricted to ≥3 weeks after the index event, likewise failed to demonstrate a significant benefit of stenting (primary outcome 8.0% vs. 7.2%; HR 1.10; *p* = 0.82), although its 30-day periprocedural event rate was numerically lower than that observed in SAMMPRIS (4, 22). Collectively, this body of evidence has restricted endovascular treatment to medically refractory cases only. More recent series, in which operators applied stricter selection criteria and delayed the procedure, have suggested a more favorable safety profile; the Wingspan StEnt System Post Market SurVEillance (WEAVE), for example, reported a 2.6% periprocedural stroke or death rate (7, 17). Our cohort recorded no periprocedural neurological complications, placing it at the favorable end of the contemporary spectrum, albeit in a limited and highly selected population. Differences in patient selection, timing from the qualifying event, device platform, operator experience, and follow-up methodology, however, substantially limit direct cross-study comparisons, and our single-arm design precludes any comparative conclusions.

Our median post-stenting residual stenosis of 21.5% is comparable to the angiographic outcomes reported in other Gateway-assisted Atlas series (9). Postprocedural residual stenosis represents more than a mere angiographic measurement and may serve as a potential predictor of long-term stent patency in patients with sICAS. In a case–control analysis of 26 patients with adequate imaging follow-up, Zhu et al. (9) demonstrated that 72.2% of patients in the non-ISR group achieved a post-procedural residual stenosis of <30%, whereas none of those who developed ISR reached this threshold (*p* = 0.014), suggesting that greater luminal gain at the index procedure is associated with a lower likelihood of subsequent restenosis. Both studies defined ISR identically (>50% stenosis within or within 5 mm of the implanted stent). We were unable to replicate this association in our cohort (Fisher’s exact test, *p* = 0.338), most likely owing to the limited number of ISR events and differences in follow-up protocols between the two series. Nonetheless, the observation that low residual stenosis may confer a protective effect against ISR warrants validation in larger cohorts.

The logic behind the single-pass approach is straightforward: once the Gateway balloon has been used for controlled submaximal angioplasty (slow inflation, typically ≤6 atm to roughly 50–70% of the reference diameter), the Atlas stent is deployed directly through the same catheter lumen, eliminating the need for intracranial microcatheter exchange. Removing this step eliminates one source of intracranial wire manipulation, which may be particularly important in tortuous or perforator-rich anatomies (23). This mechanistic point deserves particular emphasis in light of prior randomized percutaneous transluminal angioplasty and stenting (PTAS) experience. In SAMMPRIS, intraprocedural cerebrovascular events were predominantly hemorrhagic, with wire perforation–related subarachnoid hemorrhage and parenchymal hematoma contributing meaningfully to the 14.7% 30-day stroke or death rate (24). Wire perforation occurs disproportionately during distal navigation and exchange maneuvers across already-angioplastied diseased segments, where intimal disruption and vasospasm can promote wire migration into perforators or pial branches (24). By deploying the Atlas stent through the Gateway balloon catheter that already lies in stable position across the lesion, the exchange-free single-pass workflow eliminates the most hazardous re-navigation step and reduces total intracranial wire dwell time. While we observed zero periprocedural hemorrhagic complications in this cohort, the small sample size and selected, delayed-treatment population preclude any definitive conclusion that the technique reduces wire-perforation hemorrhage; however, this remains the principal *a priori* biomechanical rationale for the approach and should be tested in larger multicenter cohorts. This approach is consistent with the submaximal angioplasty principles described by Dumont et al. (25). The protective effect of slow, controlled balloon inflation against ISR was directly demonstrated by Shin et al. (26), who identified rapid balloon inflation (<30 s to nominal pressure) as an independent risk factor for ISR, with binary ISR rates of 48.3% with rapid inflation versus 7.5% with slow (~180-s) inflation (OR 57.7, 95% CI 5.5–606.8). The proposed mechanism—that rapid inflation provokes greater intimal injury, plaque rupture, and consequent neointimal hyperplasia—provides a biological rationale for our controlled submaximal protocol. Our approach is also conceptually aligned with the randomized Balloon Angioplasty for Symptomatic Intracranial Stenosis (BASIS) trial, which evaluated balloon angioplasty plus medical therapy rather than stent-assisted treatment for symptomatic intracranial stenosis (27). We cite BASIS here as part of the angioplasty-first rationale underlying controlled submaximal dilation, not as a direct safety analog for the present study (6). The Atlas is well-suited for this workflow, given its low-profile delivery system (10, 28).

### Lesion morphology and procedural performance

Lesion morphology is a well-established determinant of procedural risk and restenosis after intracranial stenting (18, 29, 30), yet has not been consistently reported across the contemporary Neuroform Atlas literature. In our cohort, Mori classification revealed that 37 of 57 lesions (64.9%) were Type C and an additional 18 (31.6%) were Type B, meaning that more than 96% of treated lesions fell into the morphologically intermediate or complex categories. Despite this high prevalence of complex anatomy, technical success was achieved in all 57 procedures, and no periprocedural neurological complications occurred. This observation supports the technical feasibility of the exchange-free single-pass Gateway–Atlas workflow even in lesions traditionally associated with elevated procedural risk, likely reflecting the combined advantages of the low-profile Atlas delivery system, controlled submaximal Gateway angioplasty, and elimination of intracranial microcatheter exchange. Furthermore, when ISR events were stratified by Mori type, a gradient emerged: 0% in Type A, 5.6% in Type B, and 13.5% in Type C lesions. While the small number of events precludes formal statistical inference, this pattern is biologically plausible—longer, more eccentric, and more angulated lesions present larger areas of intimal injury and greater shear stress heterogeneity, both of which promote neointimal hyperplasia. Notably, this Mori-based gradient parallels and partially overlaps with the basilar artery–predominant restenosis signal described below, as 13 of 18 basilar artery lesions in our cohort were Type C. Whether vessel territory or lesion morphology is the dominant driver of the observed restenosis pattern cannot be determined from this dataset, and future prospective studies should examine whether Mori classification refines vessel-specific risk stratification beyond anatomical location alone.

Comparing ISR rates across studies is challenging. We defined ISR as >50% luminal narrowing within the stent or within 5 mm of its edges, a threshold consistent with that of Zhu et al. (9), and used 3-month DSA as the primary adjudication time point. However, no universally accepted surveillance schedule for intracranial ISR exists, and the definitions vary. Large Atlas series have set the threshold at ≥70% or used different follow-up windows, which naturally yields lower ISR figures, even when the underlying biological process is identical (10, 11). In a post-hoc analysis of non-procedural events in the SAMMPRIS trial, the median time to symptomatic in-stent restenosis was 6.0 months, with an interquartile range beginning at 3.8 months, indicating that clinically meaningful restenosis can emerge relatively early after treatment (31). Therefore, a 3-month DSA evaluation may be reasonable as an early surveillance study for detecting initial restenosis patterns, even though it is unlikely to capture the full restenosis burden, which may continue to evolve over the subsequent 6 to 12 months. This interpretation is biologically plausible, as in-stent restenosis after intracranial stenting has been attributed predominantly to neointimal hyperplasia developing within the first several months after implantation, a temporal pattern documented in the early Wingspan experience (29, 30). Accordingly, our 7.1% ISR rate at 3 months should be interpreted as an early angiographic estimate rather than a definitive measure of the total restenosis burden.

Indeed, two additional ISR or occlusion events occurred in our cohort after the 3-month assessment (one delayed occlusion at month 4 and one late ISR at month 6), raising the cumulative rate to 10.5% (6/57 patients). This figure likely provides a more clinically representative estimate, although it may still underestimate the true incidence: although our institutional protocol planned a repeat DSA at 12 months for all patients, only 15 of 57 completed this examination, with the remainder followed by CTA or MRA—modalities with limited sensitivity for quantifying in-stent luminal narrowing compared with DSA. Therefore, asymptomatic or borderline restenosis beyond 3 months may have gone undetected.

The most notable exploratory signal was the BA-predominant restenosis pattern. Every case of early ISR > 50% on 3-month DSA occurred in a basilar artery lesion (4/17, 23.5% versus 0/39 in non-BA targets, *p* = 0.006), and the cumulative ISR or occlusion rate reached 29.4% in BA targets compared with 2.6% in non-BA targets (*p* = 0.008). Importantly, post-stent residual stenosis (T1) was comparable between BA and non-BA lesions (*p* = 0.215; Table 4), and within BA lesions with available 3-month DSA, post-stent residual stenosis and lesion or device dimensions were similar in lesions that did and did not develop early ISR (Supplementary Table S3). Despite adequate immediate angiographic results, the basilar artery appears to behave differently from other intracranial targets. Several vessel-specific factors may have contributed to this. The vertebrobasilar confluence has distinct hemodynamic features: basilar artery geometry influences velocity profile skewing, helical flow, and wall shear stress distribution (32), and a low wall shear stress promotes neointimal hyperplasia in stented vessels (33–35). The rich pontine perforators of the basilar artery and the concern for displacing atheroma into these branches may also discourage aggressive post-dilation (36, 37). While such a conservative approach contributes to favorable periprocedural safety, it may also leave an unstable plaque bed that is prone to restenosis. Peng et al. found that plaque burden and enhancement ratio on pre-stenting vessel wall MRI predicted target-artery stroke recurrence after Atlas stenting (11), although our dataset lacked vessel wall imaging to assess whether basilar lesions in our cohort harbored a more aggressive plaque biology.

These observations suggest that generic angiographic success criteria may be insufficient for the basilar artery and that closer imaging surveillance, including vessel wall MRI, may be warranted for posterior circulation targets after stenting (38). Notably, our finding of BA-predominant restenosis contrasts with the early US Wingspan experience, in which anterior circulation lesions (particularly the supraclinoid ICA and MCA) exhibited substantially higher ISR rates and more severe post-treatment restenosis than posterior circulation targets (29, 30). However, the Asian Wingspan registry observed a numerically opposite trend, with the highest ISR rate in the posterior circulation (36.8%) compared to the ICA (14.3%) and MCA (27.3%) (26). This geographic discrepancy raises the possibility that vessel-specific susceptibility to restenosis may be modulated by population-level differences in plaque distribution and biology, in addition to procedural and device-related factors. Given the small number of cumulative ISR or occlusion events in our overall cohort, this vessel-specific signal should be interpreted as exploratory and hypothesis-generating rather than definitive, although it may inform differentiated follow-up strategies if confirmed in future studies.

### Plaque morphology and basilar artery location

Detailed plaque morphology—including lesion length, eccentricity, angulation, and accessibility of adjacent perforating branches—was systematically captured through the retrospective Mori classification (see Methods, “Lesion morphology classification,” and Table 1). In our cohort, the majority of basilar artery lesions (13 of 18, 72.2%) were classified as Mori Type C, reflecting morphologically complex anatomy. When cumulative ISR/occlusion was stratified by Mori type, a gradient with increasing morphological complexity was observed (Type A 0%, Type B 5.6%, Type C 13.5%; Supplementary Table S4), suggesting that lesion morphology—and not vessel territory alone—may contribute to the basilar artery-predominant restenosis signal. Within the basilar artery, the five cumulative ISR/occlusion events were anatomically distributed as follows: three (60%) in the mid-basilar segment—the zone of densest pontine perforator origin—and two (40%) in the proximal (lower) basilar segment; no events occurred in the distal (upper) basilar segment near the basilar apex. This concentration of restenosis events in perforator-rich basilar segments likely reflects the conservative submaximal angioplasty approach intended to preserve perforator patency, which may in turn leave a partially residual plaque bed prone to neointimal hyperplasia under altered local hemodynamics. Vessel wall MRI, which could have provided additional plaque biology information (e.g., enhancement ratio, plaque burden), was not available in our retrospective dataset and is acknowledged as a limitation.

Patients showed clinical improvement after the procedure: the median NIHSS decreased from 4 at baseline to 2 at discharge, and the median mRS improved from 3 to 1 and remained stable at 6 months. However, this improvement cannot be attributed solely to stenting; natural recovery (particularly in the 42.1% of patients presenting with TIA), inpatient rehabilitation, and medical optimization likely contributed, and no control group was available for comparison. Despite these favorable trends, ischemic events in the target territory continued to accrue: five of 35 patients (14.3%) experienced a new TIA or stroke beyond 6 months among those who remained under follow-up. This underscores that focal revascularization does not address the underlying atherosclerotic disease and that aggressive secondary prevention must remain a priority (7). Our perioperative protocol is detailed in the Methods (“Perioperative management”) and was designed to support both procedural safety and durable secondary prevention; it consisted of elective DAPT initiation at least 5 days before the procedure, periprocedural systolic blood pressure control between 120 and 140 mmHg, and uniform high-intensity statin therapy. Our antiplatelet regimen (ticagrelor 90 mg twice daily plus aspirin 100 mg daily for 12 months, followed by aspirin monotherapy) reflects institutional practice. Ticagrelor was preferred over clopidogrel because it acts directly on the P2Y12 receptor and does not require hepatic bioactivation via CYP2C19, thereby avoiding the issue of variable clopidogrel responsiveness due to genetic polymorphisms in this enzyme (39, 40). Regimens were individualized based on clinical context and bleeding risk. While this strategy may provide more consistent platelet inhibition, the associated bleeding risks and variability in dual antiplatelet practices across centers should also be considered (7, 11).

This study had several limitations. As a retrospective single-center analysis of a carefully screened, medically refractory cohort, selection bias is a concern, and causal inferences cannot be drawn from it. There was no medical-therapy–only comparator. Because this was a single-arm cohort without a contemporaneous medical-therapy or conventional balloon-to-stent exchange comparator, our findings should be regarded as technical-feasibility and procedural-safety data rather than as evidence of clinical superiority over alternative strategies; comparative claims regarding stenting versus medical therapy or against conventional exchange workflows are not made and would require dedicated randomized comparison. Although no periprocedural neurological events occurred, the cohort size limited precision; by the rule of three, the upper 95% confidence bound for a 0/57 event rate was 5.3% (41). This statistical bound is itself clinically relevant and tempers any strong interpretation of “absence” of risk. Imaging follow-up beyond the 3-month DSA (completed by 56/57 patients) was inconsistent: CTA at 6 months was available for 43/57 patients, and among the 35 patients who reached the 12-month time point, only 15 (42.9%) underwent protocolized DSA. Because no universal ISR surveillance standard exists after intracranial stenting, our protocolized 3-month DSA provided a robust early angiographic assessment but may not have fully captured restenosis developing later in the 6- to 12-month window. The 12-month DSA subset may not be representative (survivorship and compliance bias), and symptom-driven imaging outside the protocol may introduce detection bias. Furthermore, the non-negligible cumulative target-territory ischemic event rate beyond 6 months (5/35, 14.3% among patients with available longer follow-up) reflects the natural history of underlying intracranial atherosclerotic disease, which focal revascularization does not address, and reinforces the central importance of aggressive secondary prevention. The balloon inflation duration and balloon-to-artery ratios were not systematically recorded, limiting the formal assessment of submaximal angioplasty protocol adherence. With only six ISR or occlusion events, the statistical power for predictor analyses was limited; therefore, the BA-predominant pattern should be regarded as a hypothesis rather than an established finding. The subgroup analysis (Table 4) was not adjusted for confounding because the sparse event count precluded reliable multivariable modeling within subgroups, and the observed between-group differences may therefore reflect unmeasured confounders rather than true vessel-specific effects. Mori classification was performed retrospectively by two readers in consensus; prospective grading at the time of the procedure would be methodologically preferable and is planned for future protocols at our center. Finally, this single-center experience—with most procedures performed by a single high-volume primary operator under consistent supervision—may limit external generalizability while improving internal technical consistency.

## Conclusion

In this selected retrospective single-arm cohort, the exchange-free single-pass Gateway–Atlas technique demonstrated high technical feasibility and a low observed 30-day neurological complication rate in a carefully selected sICAS cohort, which included a high proportion of morphologically complex (Mori Type B/C) lesions. This approach avoids intracranial microcatheter exchange while achieving substantial luminal gain. The absence of early neurological events should be interpreted cautiously in light of the limited sample size (upper 95% confidence bound for a 0/57 event rate, 5.3%). The observed basilar artery-predominant restenosis pattern should be regarded as exploratory and hypothesis-generating and may justify closer posterior circulation surveillance in future prospective studies.

## Data Availability

The original contributions presented in the study are included in the article/Supplementary material, further inquiries can be directed to the corresponding author.
