# Supplementary material for: Exchange-free single-pass gateway balloon–assisted neuroform atlas stenting for symptomatic high-grade intracranial atherosclerotic stenosis: clinical and angiographic outcomes
Source: Front Neurol. 2026 Jun 22;17:1854000. doi: 10.3389/fneur.2026.1854000 (PMC13333440; doi:10.3389/fneur.2026.1854000)
Supplement: Supplementary file 1 [file Supplementary_file_1.docx]

**SUPPLEMENTARY MATERIALS**

**Supplementary Figure S1**


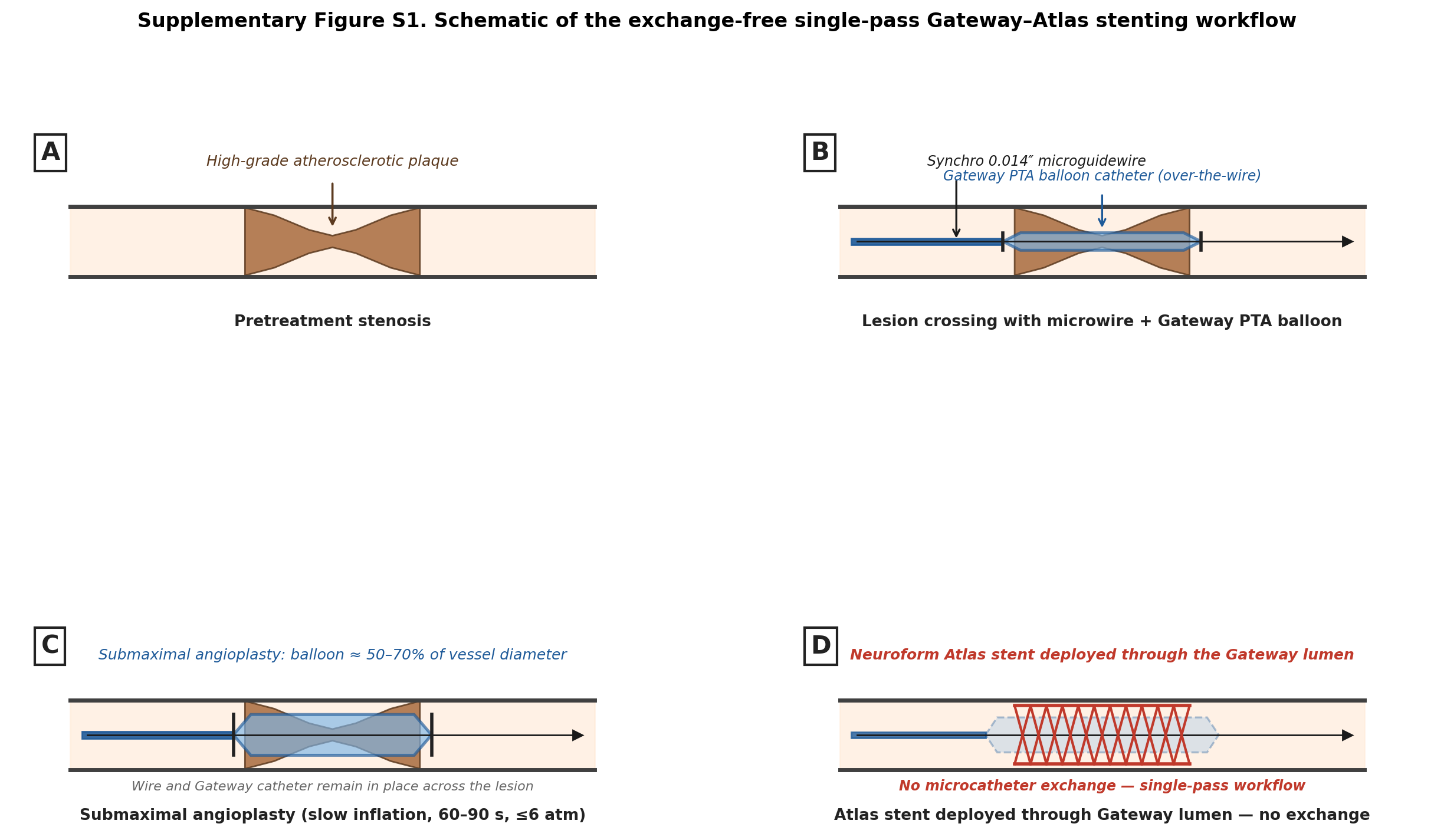


*Supplementary Figure S1. Schematic depiction of the exchange-free single-pass intracranial stenting technique. (A) Severe intracranial arterial stenosis (≥70%) due to atherosclerotic plaque. (B) Lesion traversal using a 0.014-inch hydrophilic-coated Synchro SELECT guidewire and Gateway over-the-wire (OTW) PTA balloon catheter across the stenotic segment. (C) Controlled submaximal balloon angioplasty with slow inflation (60–90 seconds, up to 6 atm) to minimize vessel trauma; the wire and Gateway catheter remain in stable position across the lesion. (D) Direct deployment of the Neuroform Atlas self-expanding stent through the Gateway balloon catheter lumen, eliminating the need for intracranial microcatheter exchange (exchange-free single-pass workflow).*

**Supplementary tables — Firth penalized logistic regression (any ISR >50%/occlusion)**

Outcome: any ISR >50% or target lesion occlusion during imaging follow-up (events=6) among patients with known ISR status (N=56). Due to the low number of events, Firth (Jeffreys-prior penalized) logistic regression was used (exploratory).

***Supplementary Table S1. Univariate Firth logistic regression for any ISR >50%/occlusion***

| **Predictor** | **OR (95% CI)** | **p** |
| --- | --- | --- |
| Age (per year) | 0.988 (0.914–1.092) | 0.786 |
| Lesion length (per mm) | 1.005 (0.873–1.076) | 0.913 |
| Atlas stent length (per mm) | 1.031 (0.936–1.108) | 0.453 |
| Male sex | 1.754 (0.313–18.111) | 0.546 |
| Hypertension | 0.218 (0.037–1.481) | 0.112 |
| Diabetes mellitus | 0.861 (0.171–5.357) | 0.860 |
| Hyperlipidemia | 4.123 (0.820–25.819) | 0.085 |
| Smoking status (reference: Never) |  |  |
| └ Never smoker | Reference | — |
| └ Current vs Never | 2.053 (0.277–15.352) | 0.465 |
| └ Former vs Never | 0.867 (0.123–6.112) | 0.880 |

***Supplementary Table S2. Parsimonious multivariable Firth model (Age + Hypertension + Hyperlipidemia)***

| **Predictor** | **Adjusted OR (95% CI)** | **p** |
| --- | --- | --- |
| Age (per year) | 0.993 (0.920–1.098) | 0.869 |
| Hypertension (yes vs no) | 0.121 (0.010–0.983) | 0.048 |
| Hyperlipidemia (yes vs no) | 6.364 (1.099–65.694) | 0.039 |

*Notes: The multivariable model was specified a priori with three covariates given the anticipated low event count. Age was retained as a baseline demographic covariate, while hypertension and hyperlipidemia were selected a priori based on their established clinical relevance to atherosclerotic disease progression. Odds ratios are exponentiated coefficients. The 95% confidence intervals are profile penalized-likelihood intervals, and p-values are from penalized likelihood ratio tests. Smoking status was parameterized with never smokers as the reference category in univariate analyses. The results should be interpreted as exploratory because of the limited number of ISR events included. Nominal statistical significance should not be regarded as confirmatory given the sparse events and extremely wide confidence intervals.*

***Supplementary Table S3. Exploratory within-basilar artery comparisons at 3-month DSA***

Basilar artery targets with available 3-month DSA (n=17) were stratified by the presence of early ISR >50% at 3 months. Values are median [IQR]. P values are from Mann–Whitney U tests (two-sided) and should be interpreted as exploratory.

| **Variable** | **No early ISR (n=13)** | **Early ISR >50% (n=4)** | **p** |
| --- | --- | --- | --- |
| Baseline stenosis, % (T0) | 88 [80–95] | 76.5 [72.8–82.5] | 0.062 |
| Post-stent residual stenosis, % (T1) | 25 [20–35] | 35 [19.5–44.8] | 0.394 |
| Lesion length, mm | 12 [10–18] | 10.5 [8.5–12.5] | 0.459 |
| Gateway balloon diameter, mm | 2.5 [2.2–2.8] | 2.8 [2.8–2.9] | 0.111 |
| Atlas stent length, mm | 24 [21–24] | 21 [21–27] | 0.406 |
| Days from index event to procedure | 10 [8–12] | 8 [8–8.5] | 0.287 |

**Supplementary Table S4. Outcomes stratified by Mori classification**

All target lesions (N=57) were retrospectively classified according to the Mori system using preprocedural digital subtraction angiography. Outcomes were stratified by Mori class for exploratory and descriptive comparison.

| **Outcome** | **Type A (n=2)** | **Type B (n=18)** | **Type C (n=37)** | **Total (n=57)** |
| --- | --- | --- | --- | --- |
| Technical success, n/N (%) | 2/2 (100) | 18/18 (100) | 37/37 (100) | 57/57 (100) |
| 30-day stroke/ICH, n/N (%) | 0/2 (0) | 0/18 (0) | 0/37 (0) | 0/57 (0) |
| Cumulative ISR >50%/occlusion, n/N (%) | 0/2 (0) | 1/18 (5.6) | 5/37 (13.5) | 6/57 (10.5) |
| Re-intervention, n/N (%) | 0/2 (0) | 0/18 (0) | 1/37 (2.7) | 1/57 (1.8) |

*Footnote: Values are n/N (%). ISR, in-stent restenosis; ICH, intracranial hemorrhage. The small number of Type A lesions limits direct statistical comparison; results should be interpreted as descriptive and hypothesis-generating. Mori classification reference: see manuscript reference [18].*

**Supplementary Table S5. Statistical details and operational definitions for Table 2**

This table consolidates the methodological notes, denominator definitions, and post-hoc statistical comparisons that were previously included as long footnotes in main-text Table 2. They have been moved here to streamline Table 2 in line with Reviewer 2's recommendation.

**Operational definitions and denominators**

• Time-window denominators reflect available follow-up at each window (3-month DSA n=56/57; 6-month clinical follow-up n=49/57; 6-month CTA n=43/57; ≥12-month follow-up n=35/57; protocolized 12-month DSA n=15/35).

• Cumulative ISR >50% / target lesion occlusion (n = 6/57, 10.5%) composed of: 4 events at protocolized 3-month DSA, 1 delayed target-lesion occlusion at month 4 (cerebellar/brainstem infarction with subsequent death), and 1 late ISR >50% at month 6 (symptomatic TIA, treated with balloon angioplasty).

• Confidence interval estimate: for the 0/57 30-day neurological complication rate, the upper 95% confidence bound is approximately 5.3% (rule of three).

• The single non-neurological 30-day mortality was sepsis on post-procedural day 24 in a patient without cerebrovascular complication.

**Functional outcome statistical comparisons**

**NIHSS (baseline vs. discharge):** Wilcoxon signed-rank test on paired observations (n=56), p<0.001.

**mRS across baseline, discharge, 3 months, and 6 months:** Friedman test on complete cases with data at all four time points (n=49), χ²=96.616, p<0.001. Bonferroni-corrected post hoc pairwise comparisons (Wilcoxon signed-rank with Bonferroni adjustment): baseline vs. discharge p_adj<0.001; baseline vs. 3 months p_adj<0.001; baseline vs. 6 months p_adj<0.001; discharge vs. 3 months p_adj=0.027; discharge vs. 6 months p_adj=0.800; 3 months vs. 6 months p_adj=1.000.

*Abbreviations: CTA, computed tomography angiography; DSA, digital subtraction angiography; ICH, intracranial hemorrhage; ISR, in-stent restenosis; mRS, modified Rankin Scale; NIHSS, National Institutes of Health Stroke Scale; TIA, transient ischemic attack.*
